# The Effect of Glucagon-Like Peptide-2 Receptor Agonists on Colonic Anastomotic Wound Healing

**DOI:** 10.1155/2010/672453

**Published:** 2010-10-04

**Authors:** Heather A. Redstone, William D. Buie, David A. Hart, Laurie Wallace, Pamela J. Hornby, Sarah Sague, Jen J. Holst, David L. Sigalet

**Affiliations:** ^1^Division of Colorectal Surgery, Department of Surgery, Faculty of Medicine, University of Calgary, Calgary, AB, Canada T2N 1N4; ^2^McCaig Institute for Bone and Joint Health, Department of Surgery, Faculty of Medicine, University of Calgary, Calgary, AB, Canada T2N 1N4; ^3^Gastrointestinal Research Group and Division of Pediatric General Surgery, Department of Surgery, Faculty of Medicine, University of Calgary, Calgary, AB, Canada T2N 1N4; ^4^Clinical Research and Development, Centocor Inc., Radnor, PA 19087-4557, USA; ^5^Panum Institute, University of Copenhagen, 1165 Copenhagen, Denmark; ^6^Department of Surgery, Alberta Children's Hospital, 2888 Shaganappi Trail NW, Calgary, AB, Canada T3B 6A8

## Abstract

*Background*. Glucagon-like peptide 2 (GLP-2) is an intestinal specific trophic hormone, with therapeutic potential; the effects on intestinal healing are unknown. We used a rat model of colonic healing, under normoxic, and stress (hypoxic) conditions to examine the effect of GLP-2 on intestinal healing. *Methods*. Following colonic transection and reanastomosis, animals were randomized to one of six groups (*n* = 8/group): controls, native GLP-2, long-acting GLP-2 (GLP-2- MIMETIBODY, GLP-2-MMB), animals were housed under normoxic or hypoxic (11%  O_2_) conditions. Animals were studied five days post-operation for anastomotic strength and wound characteristics. * Results*. Anastomotic bursting pressure was unchanged by GLP-2 or GLP-2-MMB in normoxic or hypoxic animals; both treatments increased crypt cell proliferation. Wound IL-1*β* increased with GLP-2; IFN*γ* with GLP-2 and GLP-2-MMB. IL-10 and TGF-*β* were decreased; Type I collagen mRNA expression increased in hypoxic animals while Type III collagen was reduced with both GLP-2 agonists. GLP-2 MMB, but not native GLP-2 increased TIMP 1-3 mRNA levels in hypoxia. *Conclusions*. The effects on CCP, cytokines and wound healing were similar for both GLP-2 agonists under normoxic and hypoxic conditions; anastomotic strength was not affected. This suggests that GLP-2 (or agonists) could be safely used peri-operatively; direct studies will be required.

## 1. Introduction

Many patients require surgical resection of the small or large bowel for a wide range of indications, including colorectal cancer, inflammatory bowel disease, and trauma. Leakage from such intestinal anastomoses is a major source of morbidity and mortality in gastrointestinal surgery [1]. Anastomotic dehiscence not only leads to peritonitis and sepsis but is also associated with increased rates of local recurrence of carcinoma [2]. While the overall rate of anastomotic leakage in colorectal surgery is between 3.4 and 6%, the mortality from anastomotic leaks following low anterior resection ranges between 6.0 and 39.3% [3]. Thus any potential perioperative treatment must be closely evaluated for its effect on healing.

Anastomotic healing is a complicated series of events which proceeds along a course similar to that of the more familiar skin wound, but at an accelerated rate [4–7]; intestinal wounds have regained their previous full strength by 7–10 days. Alterations of cytokine or growth factor expression, quantitatively, qualitatively, or temporally, can have an effect on subsequent wound healing [8, 9]. Agents which have shown positive effects in experimental studies include growth hormone [5, 10] and insulin-like growth factor-1 [11, 12]. However the systemic effects of these agents limit their clinical usefulness for this indication.

Glucagon-like peptide-2 (GLP-2) is a 33-amino acid peptide with structural similarity to glucagon. It is an enteroendocrine hormone secreted by the L-cells in the mucosa of the small intestine and colon and acts primarily as a regulator of nutrient absorption and an intestinal specific trophic factor [13–15]. Interestingly, the widespread effects of GLP-2 in the intestine are mediated by indirect pathways; the receptor is not expressed on the epithelium but on enteric neurons, scattered enteroendocrine cells, and the pericryptal myofibroblast [16–20]. In the short term, GLP-2 increases intestinal blood flow [18] while over the longer term in noninflamed tissue it specifically increases crypt cell proliferation (CCP), with subsequent increases in crypt depth and villus height and a net increase in mucosal nutrient transport [14, 21]. However, in inflamed mucosa, GLP-2 is antiproliferative, decreasing the expression of proinflammatory cytokines while increasing the expression of IGF-1 with the global effect of increasing the healing of inflamed mucosa [19, 20]. These effects are not mediated through IL-10 or TGF-*β* expression [20]. Of interest, the intestinotrophic effects of GLP-2 are proposed to be mediated through IGF-1 expression [22], and IGF-1 has been shown to enhance anastomotic wound healing with improved bursting strength and increased collagen deposition at the anastomosis [11, 12, 23, 24]. From this, we theorized that GLP-2 may have the same wound healing benefits as IGF-1 but without its systemic effects. Alternatively, GLP-2 could potentially impair healing through its antiinflammatory effects, as has been shown with other anti-inflammatory agents such as corticosteroids and nonsteroidal anti-inflammatory drugs [25–27].

Glucagon-like peptide-2- mimetibody construct (GLP-2-MMB) is a long-acting form of GLP-2. The human GLP-2 (1-33) long acting analogue (an arginine to glycine substitution of the second amino acid at the N-terminus) is coupled to a domain that includes the Fc portion of an antibody, in this case a rat IgG2b Fc isotype with a human IgG4 hinge [28]. The increased molecular weight and pharmacokinetic properties of the Fc construct dramatically increase the half-life compared to engineered native peptides which may be useful therapeutically [28–30]. 

We hypothesized that GLP-2 would have a positive effect on the healing anastomosis by increasing the production of anti-inflammatory cytokines IL-10 and prohealing TGF-B, with no change in the pro-inflammatory cytokines IL-1*β* *α* and IFN-*α*. We compared the effect of the long-acting receptor agonist GLP-2-MMB with native GLP-2 using different states of systemic oxygenation to mimic clinical perioperative stressors [6, 31]. A human study has shown that a perianastomotic oxygen tension of <20 mmHg was highly predictive of anastomotic leakage; in this study ambient oxygenation levels were set so that tissue PO_2_ levels were 40–50 mm Hg [6, 31]. The time at which leaks appear is typically from days 5 to 7 post operation, which is the phase of maximal collagenase activity in the wound; accordingly the fifth day postoperation was chosen as the study endpoint [3, 4, 32]. 

## 2. Methods

### 2.1. Animals and Surgical Procedure

 Ethical approval for the experimental protocol was granted by the Animal Care Committee of the Faculty of Medicine, University of Calgary. Male Sprague-Dawley rats (250–300 g) were acclimatized for a week and fasted for 24 hours prior to surgery with free access to water. Prior to surgery, animals were randomized to the GLP-2, the GLP-2-MMB, or control groups. Animals were injected s.c. one hour prior to surgery with 100 *μ*g/kg (1-33) GLP-2 (American Peptide, Sunnyvale California) in 1ml normal saline, 2 mg/kg GLP-2-MMB (Centocor Inc R. and D., Radnor PA) diluted in 1ml of normal saline, or 1 ml of normal saline (controls). GLP-2-MMB dosing was based on previous studies, establishing dose-response equivalence for increasing CCP with native GLP-2 [28]. Animals were weighed and anesthetized using 1-2% isoflurane. A prophylactic dose of 25 mg cefazolin (Novopharm Limited, Toronto, ON, Canada) was given subcutaneously just prior to surgical incision. The transection-anastomosis was done in the midportion of the transverse colon, following our previously described methods [6]. The marginal vessels were ligated with 6-0 silk (Ethicon Inc., Somerville, NJ), the bowel was transected and an anastomosis created with 10–14 interrupted 6-0 monofilament polypropylene sutures (Proline, Ethicon Inc. Somerville, NJ). The abdomen was irrigated with saline and closed in two layers (skin and muscle) using 4-0 Vicryl running sutures (Ethicon). A subcutaneous fluid bolus of 10 ml of normal saline was given, and a single intramuscular dose of 0.015 mg buprenorphine (Temgesic, Schering-Plough Ltd., Hertfordshire UK) was given for analgesia.

### 2.2. Treatment Groups

Following surgery, the animals were randomized a second time to be placed in a normoxic (FiO_2_ = 21%) or hypoxic (FiO_2_ = 11%) environment, following our previously described methods [6]. In brief, an airtight lid was fitted to a standard polyethylene rat cage, with appropriate connections for controlled air input. The hypoxia generator (Hypoxico Inc, New York) was set on 50% which gave a hypoxic environment of approximately 11%  FiO_2_, corresponding to a tissue oxygenation of 40–50 mm Hg [6]. The oxygen content of the chamber was measured daily using a MiniOx I oxygen analyzer (MSA Medical Products, Pittsburg, PA). Normoxic animals were maintained in standard rat cages. All rats were housed in pairs, and food intake was recorded over the course of the study period. Animals were pair-fed a standard rodent pellet diet and allowed water *ad libitum*. A total of 48 animals were used for the experiment and were divided into six treatment groups: normoxic control, normoxic GLP-2, normoxic GLP-2-MMB, hypoxic control, hypoxic GLP-2 and hypoxic GLP-2-MMB. Animals were maintained in their normoxic or hypoxic environments, for the duration of the experiment (5 days). All animals were injected twice daily, from 0800 to 0900 and again at 1700 to 1800 controls with saline, GLP-2, animals with 100 *μ*g/kg/day (1-33) GLP-2, GLP-2-MMB animals with 2 mg/kg of the GLP-2-MMB on days 0 and 3 and saline at the other time points. Each injection lasted less than 90 seconds, and the animals were returned immediately to the appropriate oxygen environment.

### 2.3. Endpoints

 On postoperation day five, with continuation of a normal feeding pattern between 9 and 11 am, coincident with the treatment injection (saline/GLP-2 ligand), animals were injected intraperitoneally with 100 mg/kg of 5-Bromo-2′-deoxyuridue (BrdU) (Sigma-Aldrich Inc, St. Louis MO, USA). One hour post saline/GLP-2/MMB and BrdU injection, they were anesthetized with 1-2% isoflurane and weighed, and a laparotomy was performed. Approximately 5 ml of blood was taken by cardiac puncture drawn into iced EDTA vacutainers containing 10% volume of 5000 KU trasylol and 0.1 mM diprotinin A, centrifuged within one half hour, and the serum was frozen. GLP-2 (1-33) quantification was performed using a RIA specific for the active N-terminus of 1-33 GLP-2 (J. J. Holst, Panum Institute, Copenhagen, Denmark, antibody no. 92160) [33]. This does not capture the GLP-2 activity of the MMB construct since the antibody precipitates during the sample preparation [33]. 

The animals were then euthanized by exsanguination. The entire length of bowel from pylorus to distal colon was removed. The length and width of small and large intestine were measured, under standardized tension (2 g). The colon was then divided 3 cm proximally and distally to the anastomotic sites. Any adhesions to the anastomosis were removed *en bloc* with the segment of colon to preserve the integrity of the anastomosis.

### 2.4. Burst Pressure Measurements

 The bursting pressure was measured using an infusion pump coupled to an in-line sphygmomanometer (Welch Allyn Tycos, Skaneateles Falls, NY), as previously described in [6]. The bowel was submerged in a saline bath and infused with air at a rate of 5 ml/minute. Bursting pressure was recorded as the pressure where visualization of bubbles was first observed.

### 2.5. Tissue Preparation

 The ties were removed, and the segment of bowel containing the anastomosis was opened longitudinally. A segment containing the anastomosis and 5 mm of colonic tissue on either side was removed. It was divided longitudinally into three segments: one for histology, one for cytokine analysis, and one for RNA isolation for RT-PCR and processed as described below. We have shown previously that the process of determination of bursting pressure does not change histological scores, cytokine or mRNA content of the tissue [6].

### 2.6. Collagen, Tissue Inhibitors of Metalloproteinases, and Collagenase Analysis

 The primary strength within the intestinal wound comes from collagen I and III; the increase in collagenase 13 activity over days 4–8 is thought to contribute to the development of leakage, especially with coexisting inflammation [34, 35]. Tissue inhibitors of metalloproteinases (TIMPs) counteract this activity [36]. To examine the effects of GLP-2 therapy on these factors, the mRNA levels for each molecule were assessed in the perianastomotic tissue. As described, a longitudinal segment of bowel tissue containing the anastomosis and 5 mm of bowel on either side was flash frozen in liquid nitrogen and then stored at −80°C until processing. Total RNA was extracted using the TRIspin method [37] and quantified using the SYBR Green reagent (Molecular Probes, Eugene, OR) method. Simultaneous reverse transcription (RT) reactions using 1ug of RNA from all samples in each group were carried out with the OmniScipt kit (Qiagen, Hilden, Germany). Polymerase chain reaction (PCR) was used to assess mRNA levels. Primers for collagen type I, type III, TIMP 1, 2, and 3, and matrix metalloproteinase 13 (MMP-13) were synthesized on site; sequences were as reported previously in [36, 38]. PCR conditions were optimized and rigorously controlled for each molecule (temperatures, number of cycles) to ensure detection was in the linear phase of the amplification. Agarose gel electrophoresis followed by staining with ethidium bromide was used for separation and detection of the PCR-generated cDNA amplicons (Gel Doc XR System; BioRad, Hercules, CA). The results were normalized by dividing values for each gene to the expression of *β*-actin in the same sample; the expression of *β*-actin, a housekeeping gene, does not change at the anastomosis [6]. Reanalysis for a subset of the genes assessed with a second aliquot of RNA revealed nearly identical results to those reported Furthermore, in other systems, a direct comparisons between the mRNA analysis using the indicated methodology and real-time qPCR has revealed nearly identical results for the high copy number genes assessed (Hart DA and Reno CR, unpublished).

### 2.7. Cytokine Analysis

 Tissues were stored at −80°C until processing. Perianastomotic specimens as detailed above were thawed, washed in saline, and then homogenized with 5 volumes of homogenizing solution. The homogenizing solution was made of 50 ml 100 mM phosphate buffered saline (PBS), pH 7.0, with one complete protease inhibitor cocktail tablet (Roche Diagnostics, Mannheim, Germany). The tissue was homogenized in a Polytron homogenizer (Kinematic model 2100, Switzerland). Homogenates were centrifuged at 2000 rpm for 20 minutes to remove large tissue particles and then centrifuged at 13, 200 rpm for 60 minutes to obtain a clear supernatant. The supernatant was aliquoted and stored at −80°C until further use. Protein content from the homogenate for each specimen was determined according to Lowry's method [39].

The supernatants were quantitatively assayed using enzyme-linked immunosorbent assay (ELISA) kits for rat tumor necrosis factor-*α* (TNF-*α*) (Assay Design, Ann Arbor, Michigan, USA) interleukin 1-*β* (IL-1*β*), interleukin-10 (IL-10), interleukin-13 (IL-13) transforming growth factor-*β* (TGF-*β*), interferon *γ* (IFN*γ*) (Biosource, Camarillo, California, USA), each following the manufacturer's directions. All specimens were analyzed in duplicate, and then normalized to the total protein content of the sample, and expressed as pg/mg protein.

### 2.8. Crypt Cell Proliferation

 As a measure of the biological effect of each GLP-2 receptor ligand, crypt cell proliferation was measured. At the time of euthanasia, a segment of distal ileum, approximately 10 cm from the ileocecal valve, was harvested and preserved in 10% formalin for at least 48 hours. The tissue was then dehydrated and embedded in paraffin blocks. Sections 6 *μ*m thick were cut using a microtome, and immunohistochemical staining was done for BrdU-(antibody 1 : 100 rabbit anti-BrdU; Serotec, London, UK). The number of BrdU stained cells the total cells per half crypt were then counted in five crypts for each animal, and expressed as the ratio of BrdU + cells/total cells per half crypt, and averaged to give the value for each animal [19]. 

### 2.9. Histology

A strip of tissue containing the anastomosis was placed immediately into 10% formalin and preserved for at least 48 hours and then dehydrated and embedded in paraffin blocks. Sections 6 *μ*m thick were cut using a microtome and stained with hematoxylin and eosin (Sigma, St. Louis, MO, USA) and examined using standard light microscopy. All observations were made by one examiner (H.A. Redstone) who was blinded to the treatment group. Sections were evaluated using a semiquantitative scoring method modified from our previous report [6, 40]. In brief, each of the parameters of healing, inflammatory reaction, submucosal/muscularis bridging, and mucosal epithelial healing, were assigned a score from 0 to 3, with 0 being normal and the maximum total of nine with a lower score indicating improved healing.

### 2.10. Statistical Analysis

 Results are expressed as mean ± standard error of the mean. Significance differences were assessed using the one-way ANOVA with Tukey's post test analysis. *P* values of <.05 were considered statistically significant. The computer software program GraphPad Prism version 4.01 (Prism Corp, La Jolla, CA) was used for these calculations.

## 3. Results

### 3.1. Clinical Outcome and Gross Morphology

 All animals tolerated surgery, with two perioperative deaths, one in the normoxic control group on day one and one in the hypoxic GLP-2-MMB group on the fourth postoperative day. The anastomoses of these two animals were intact on necropsy, and no specific cause could be identified; these results were excluded from further analysis. There were no differences in change in body weight, food consumption, and small or large bowel length and width with GLP-2 and GLP-2-MMB treatment as compared to controls in either the normoxic group or hypoxic group (data not shown).

### 3.2. Crypt Cell Proliferation

**Figure 1:**
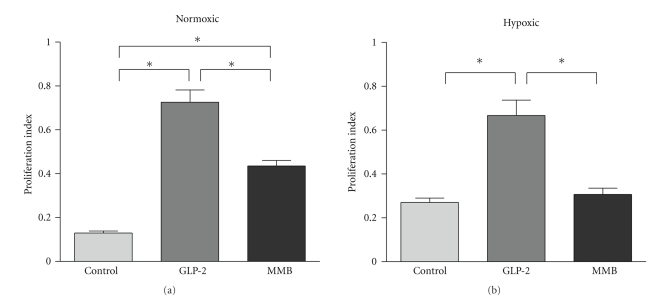
Crypt cell proliferation index. Proliferating cells in the ileal crypts were detected by BrdU immunohistochemical labelling; the index is the number of labelled cells per total cells in each half crypt, averaged from 5 or more crypts per animal, *n* = 5 or more per group. Data: mean ± SEM, *P < .05 versus the group indicated, †*P* < .05 versus similarly treated group under normoxic conditions. Comparisons by ANOVA.

 Crypt cell proliferation (CCP) was significantly increased in both the GLP-2 and GLP-2-MMB-treatment groups (Figure 1) under normoxic conditions. There was an expected increase in CCP in control animals under hypoxic conditions; under these conditions, the proliferation rate of the MMB-treated animals did not increase (Figure 1) [6]. The native GLP-2 retained a proliferative effect under hypoxic conditions.

### 3.3. Bursting Pressure Assessment

**Figure 2:**
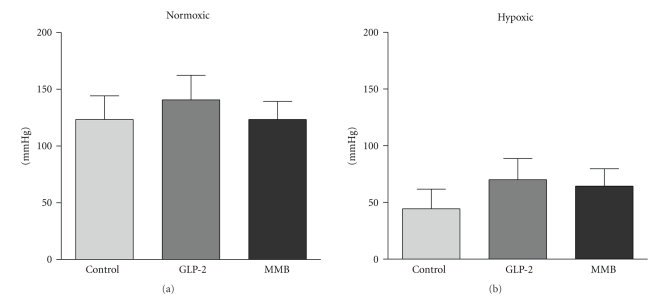
Anastomotic bursting pressures of normoxic and hypoxic animals. Groups treated with GLP-2, GLP-2-mimetibody (MMB) and controls. Isolated segments of intestine insufflated with air under saline: pressure at which the first bubbles appear is recorded. (*n* = 7 or more per group, Data: mean ± SEM, *P < .05 versus similarly treated group under normoxic conditions. Comparisons by ANOVA).

 As expected, anastomotic bursting pressures were significantly lower in the hypoxic animals but there were no differences with either GLP-2 or GLP-2-MMB treatment within the hypoxic or normoxic groups (Figure 2).

### 3.4. Histological Analysis

**Figure 3:**
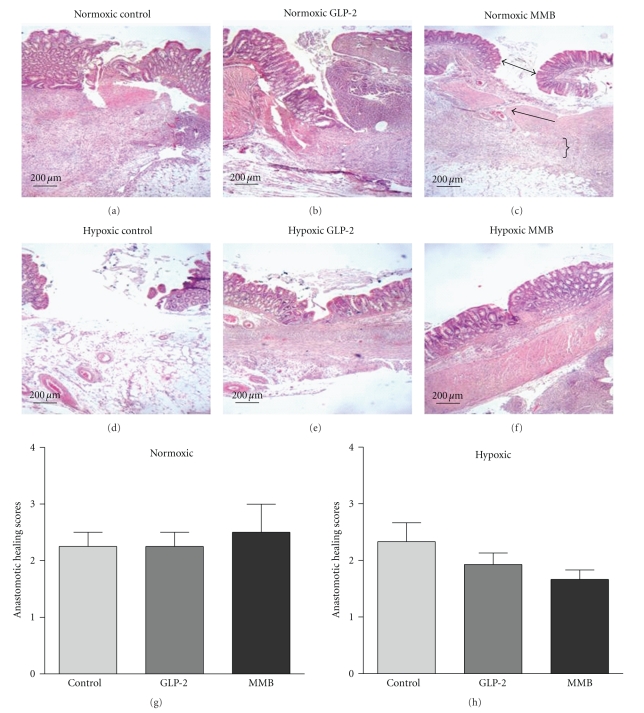
Histological sections of colonic anastomosis (H and E × 4) ({indicates zone of inflammation, arrows; indicate zones of epithelial and muscular bridging}). Anastomotic healing scores in normoxic and hypoxic animals: treated with GLP-2, GLP-2 mimetibody (MMB) and controls. Rating scale 0–9, with 0 indicating perfect healing [5]. data: mean ± SEM; *n* = 7 or more per group.

 The mean anastomotic healing scores for the groups were not significantly different with either GLP-2 or GLP-2 mimetibody treatment as compared to controls, under either normoxic condition or hypoxic condition (Figure 3). There were areas of considerable artifact due to the disruption of tissue planes by the bursting pressure studies; however, the status of the surface epithelium, muscular layer, and inflammation in the subserosa could be determined in all animals (Figure 3).

### 3.5. GLP-2 Serum Levels

 Mean serum levels of GLP-2 were 35.0 ± 3.1 in control animals (similar in both normoxic and hypoxic), 119 ± 32 in GLP-2-treated animals, and 49.1 ± 10.3 pmol/l in GLP-2-MMB-treated animals. There were no differences between the levels in hypoxic and normoxic animals. The serum levels of GLP-2 were significantly higher in animals receiving GLP-2 (1-33) (*P* < .05); however, the levels in GLP-2-MMB-treated animals were not significantly elevated compared to controls. Importantly, the levels in GLP-2 MMB animals reflect only endogenous GLP-2, since the assay does not detect antibody bound activity [33].

### 3.6. Cytokine Analysis

**Figure 4:**
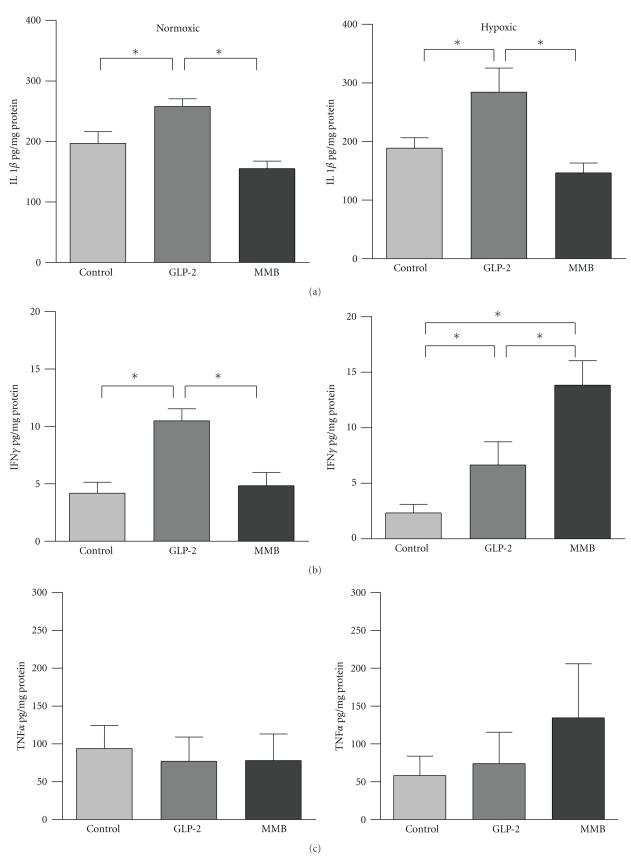
Pro-inflammatory cytokines: IL1*β* (a), IFN*γ* (b) and TNF*α* (c) as measured by ELISA and normalized to protein content in anastomosed colon from normoxic and hypoxic animals treated with GLP-2, mimetibody or control. Data: mean ± SEM, *n* = 7 or more per group, *P < .05 by ANOVA.

**Figure 5:**
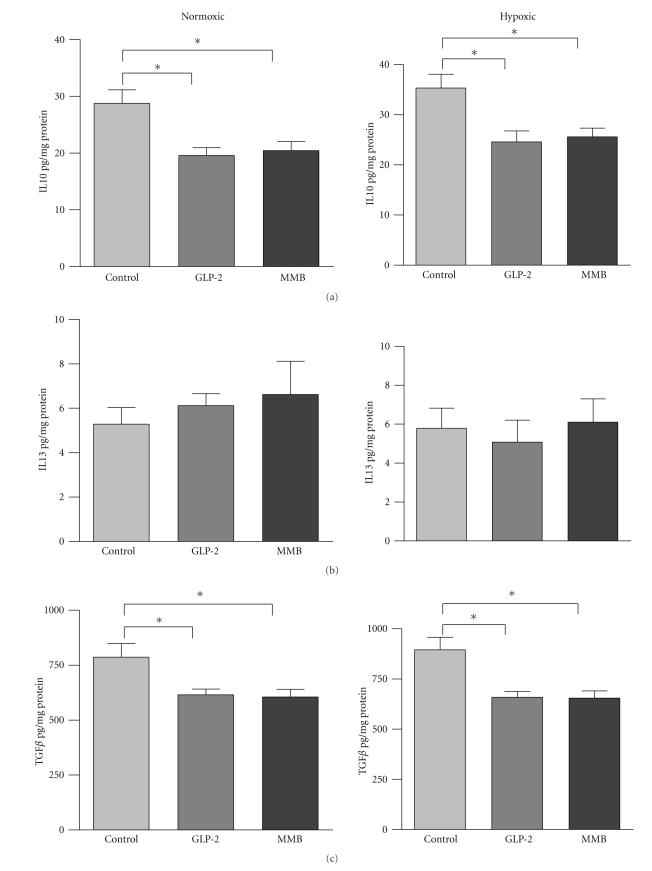
Levels of anti-inflammatory/prohealing cytokines. (a) IL-10, (b), IL-13, and (c) TGF*β* from anastomosed colon as determined by ELISA and normalized to protein content. Data: mean ± SEM, *n* = 7 or more per group, *P < .05 by ANOVA.

Levels of the pro-inflammatory cytokines IL-1*β*, TNF-*α*, and IFN-*γ* in GLP-2-treated and control animals are shown in Figure 4. Levels of IL-1*β* and IFN-*γ* were both increased with GLP-2 under both normoxic and hypoxic conditions. GLP-2-MMB treatment resulted in a decrease in IL-1*β* under both oxygen conditions and resulted in a differential effect on IFN*γ* levels; these were decreased under normoxic and increased under hypoxic conditions. Levels of TNF-*α* were not significantly affected by either form of GLP-2. Levels of the anti-inflammatory cytokine IL-10 as well as TGF*β* and IL-13 are shown in Figure 5; GLP-2 and GLP-2-MMB treatment resulted in a significant decrease in mucosal IL-10, but no change in IL-13 levels. TGF-*β* levels were decreased by both GLP-2 therapies, in both hypoxic and normoxic animals.

### 3.7. Collagen, Tissue Inhibitors of Metalloproteinases, and Collagenase Expression

**Figure 6:**
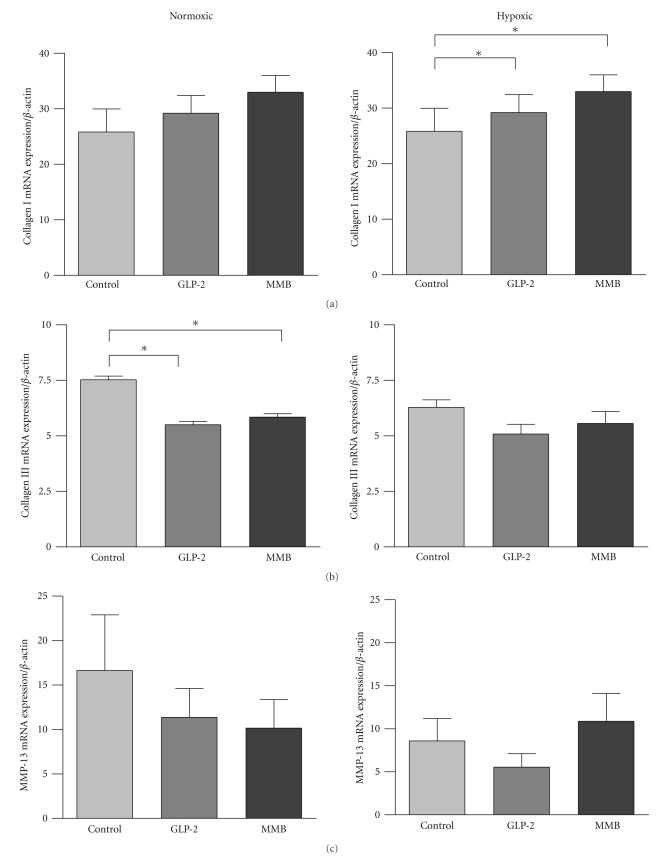
RT-PCR analysis of collagen and collagenase (MMP-13) mRNA Levels. mRNA expression of collagen I (a), collagen III (b), and matrix metalloproteinase 13 (c) in anastomotic tissue, as measured by RT-PCR normalized to *β*-actin. Data: mean ± SEM, *n* = 7 or more per group, *P < .05 versus the group indicated, by ANOVA.

 Results of RT-PCR assessment of mRNA levels for collagen types I (cI) and III (cIII) and MMP-13 are shown in Figure 6. Expression of cI mRNA in hypoxic conditions was increased by both GLP-2 receptor agonists. Levels of cIII mRNA were decreased under normoxic conditions by both GLP-2 agonists, while the changes under hypoxic conditions were not significant. There were no major effects on MMP-13 mRNA levels by the GLP-2 agonists, under either ambient oxygen status; however, there were interesting statistically significant increases in mRNA levels for TIMP 1, 2, and 3 in hypoxic animals treated with GLP-2-MMB, but not native GLP-2 (data not shown).

## 4. Discussion

This study is the first to examine the effects of GLP-2 on intestinal anastomotic healing. It evaluates the effect of GLP-2 on colonic anastomosis in an animal model of normal and impaired wound healing (hypoxia) using both native GLP-2 as well as a long-acting form of the hormone, GLP-2-MMB. Our original hypothesis was that healing would be improved with the addition of GLP-2, especially in hypoxic animals; this did not occur. However, this study did reveal several important findings. First, the results indicate that exogenous GLP-2 does not exert a negative effect on anastomotic integrity. Secondly, exogenous GLP-2 affects both pro- and anti-inflammatory pathways within the healing anastomosis and also effects collagen gene expression, altering the types of collagen that are deposited in the wound. Finally, it confirms that the long acting GLP-2 mimetibody does have biological activity similar to native GLP-2; however, the dosing may not result in a biologically equivalent effect. 

Anastomotic bursting pressure has long been used as a validated measure of anastomotic strength [6, 24, 25, 41]. This study shows no difference in bursting pressure with GLP-2 treatment, under normal or stressed (hypoxic) conditions. Thus GLP-2 does not seem to alter the strength of anastomotic healing; this finding does have clinical implications. GLP-2 is presently being investigated for therapeutic application in inflammatory bowel disease and short bowel syndrome. Patients with these disorders often require surgical resection of the gastrointestinal tract; therefore, based on these data which tested anastomotic integrity at the phase of maximal susceptibility (5 days post surgery in this model), concurrent GLP-2 therapy should not affect either the timing of surgery or anastomotic integrity. 

These results do provide additional insights into the effects of GLP-2 in the dynamic milieu of the healing anastomosis. In the gastrointestinal tract the major pro-inflammatory cytokines include IL-1*β*, TNF-*α*, and IFN-*γ* and the anti-inflammatory cytokine IL-10, while TGF-*β* is a major regulator of healing. Inflammation is an integral component of wound healing, and the pro-inflammatory cytokines are important to this process. Surgical trauma initiates an acute inflammatory response that includes the release of a number of cytokines including IL-1*β*, TNF*α*, and IFN*γ*. Following the early post wounding response, there is a shift in the cytokine profile from pro-inflammatory to anti-inflammatory/prohealing cytokines, including IL-10, IL-13, and TGF-*β* [5, 34]. The anti-inflammatory cytokines limit the duration and magnitude of the inflammatory response which allows the wound to evolve into the proliferative phase of healing. This shift from an inflammatory environment to a healing environment is coordinated by the wound macrophage, likely under the direction of cytokine signals. It has been shown that both IFN*γ* and TNF*α* are key mediators that influence macrophage expression [42]. Our previous work has also shown that in models of intestinal inflammation, GLP-2 reduces the mucosal content of inflammatory cytokines and TGF-*β* but increases the macrophage production of IGF-1 [19, 20]. In the present study, with a different inflammatory stimulus, native GLP-2 treatment resulted in increased levels of the proinflammatory cytokines IL-1*β* and IFN*γ* and decreased amounts of the anti-inflammatory cytokines IL-10 and prohealing factor TGF-*β* and yet healing appeared to proceed normally (Figures 4 and 5). GLP-2-MMB showed a different profile in the inflammatory cytokines with a decrease in IL-1*β* under both normoxic and hypoxic conditions, and a further increase in IFN-*γ* under hypoxia. The sum of these findings is that these cytokine effects were not sufficient to change wound healing, this may be because the relative levels are many times greater than baseline, and that all levels seen were adequate to stimulate a vigorous healing response [5]. A limitation of the study was that we were not able to quantify the levels of IGF-1 at the anastomosis; this may be an important link between the varying effects of GLP-2 noted under different conditions [5, 12]. 

The lower levels of IL-10 and TGF-*β* following GLP-2 treatment (with both ligands) noted in this study were similar to the results seen in previous studies, and this suggests that any effects GLP-2 may be exerting in the inflamed tissue are not likely due to an increase in these factors (Figure 5) [19, 20]. In addition, the current study examined the healing anastomosis at day five, which was chosen as a time interval when intestinal wound healing is transitioning from the inflammatory phase into the proliferative phase. Previous studies assessing inflammatory cytokines in healing anastomotic wounds have shown an early peak on the first postoperative day with a subsequent drop by day 5; thus the present study may have missed some important early changes in wound healing signals; further investigation of the temporal sequence of these events is warranted [5, 34]. 

Collagen synthesis is one of the most fundamental steps in anastomotic healing. Collagen rich tissue laid down by fibroblasts and intestinal smooth muscle cells replaces the provisional matrix that was established during the inflammatory phase. A small disturbance in the balance between collagen synthesis, deposition, cross-linking, and degradation may result in defective wound healing [7]. In the present study we show that GLP-2 treated animals exhibited modest increases in mRNA levels for type I and reduced mRNA levels for type III collagen, which was also affected by the ambient oxygen concentration (Figure 6). Collagen I is the most frequent collagen in bowel tissue and collagen III is the second most common [43]. Clinical studies have shown decreased collagen I and III expression in patients with anastomotic leakage after colorectal surgery [35]. The effects of GLP-2 and GLP-2 MMB were reassuring. There was no effect on collagen I mRNA under the normoxic conditions, but there was a decrease in collogen III; under the stress of hypoxia collagen I mRNA levels were increased by both ligands (Figure 6). The GLP-2 ligands showed no effects on mRNA levels for the TIMPs, except for an increase under hypoxic conditions with GLP-2 MMB (data not shown), and MMP-13 expression was not changed but trended downward (Figure 6). In aggregate, these findings would suggest a slight increase in collagen I synthesis with either GLP-2 ligand and, interestingly, a distinct increase in TIMP activity with the long-acting GLP-2 MMB under the stress of hypoxia. The finding of a modest (but not significant) increase in the bursting pressures in the hypoxic animals treated with the GLP-2 agonists suggests that the overall strength of the wound is improved by the agents (Figure 2). In order to fully appreciate the effects of these changes on collagen metabolism in the wound it would be useful to directly measure the levels of collagen 1 and 3; however, because of the small quantities available this was not possible in the current protocol but would be useful in future studies. 

From a therapeutic viewpoint, GLP-2 is limited by its short half-life, thus GLP-2-MMB is an attractive alternative. Anastomosis treated with GLP-2 and GLP-2-MMB demonstrated similar cytokine profiles, and the biological effects of GLP-2 and GLP-2-MMB were similar as measured by crypt cell proliferation (Figure 1). The lesser effect of the MMB ligand suggests that despite the long half-life there was a pharmacodynamically relevant reduction in activity; this could be compensated for by increasing the dose, and the frequency of administration until the crypt proliferation is equivalent to the relevant index dose of GLP-2 [44]. The relatively high GLP-2 levels in the controls are likely due the conditions of the testing; animals were not fasted and typically ate in the predawn hours, increasing the release of endogenous GLP-2 [15]. Importantly, there was no difference with respect to anastomotic integrity when compared to the native peptide. Thus GLP-2-MMB appears to be a viable alternative to the native peptide, with a convenient long dosing schedule, but preserved biological effects. 

In summary, we have shown that GLP-2 alters the cytokine profile through both the inflammatory and anti-inflammatory cascade and collagen gene expression in both normoxic and hypoxic anastomoses. However this does not translate into a change in anastomotic strength. From a clinical point of view, GLP-2 does not exert a negative effect on the healing anastomosis. Therefore, patients receiving GLP-2 agonist therapeutically who require surgery should not have impaired anastomotic healing, however, direct study will be required in the future.
